# Physical Layer Security Using Two-Path Successive Relaying

**DOI:** 10.3390/s16060846

**Published:** 2016-06-09

**Authors:** Qian Yu Liau, Chee Yen Leow, Zhiguo Ding

**Affiliations:** 1 Wireless Communication Centre, Faculty of Electrical Engineering, Universiti Teknologi Malaysia, Skudai 81310, Johor, Malaysia; qianyuliau@gmail.com; 2 School of Computing and Communications, Lancaster University, Lancaster, LA1 4YW, UK; z.ding@lancaster.ac.uk

**Keywords:** wireless sensor network, 5G, physical layer secrecy, cooperative relay networks, two-path successive relaying, secrecy capacity, intercept probability, secrecy outage probability

## Abstract

Relaying is one of the useful techniques to enhance wireless physical-layer security. Existing literature shows that employing full-duplex relay instead of conventional half-duplex relay improves secrecy capacity and secrecy outage probability, but this is at the price of sophisticated implementation. As an alternative, two-path successive relaying has been proposed to emulate operation of full-duplex relay by scheduling a pair of half-duplex relays to assist the source transmission alternately. However, the performance of two-path successive relaying in secrecy communication remains unexplored. This paper proposes a secrecy two-path successive relaying protocol for a scenario with one source, one destination and two half-duplex relays. The relays operate alternately in a time division mode to forward messages continuously from source to destination in the presence of an eavesdropper. Analytical results reveal that the use of two half-duplex relays in the proposed scheme contributes towards a quadratically lower probability of interception compared to full-duplex relaying. Numerical simulations show that the proposed protocol achieves the ergodic achievable secrecy rate of full-duplex relaying while delivering the lowest probability of interception and secrecy outage probability compared to the existing half duplex relaying, full duplex relaying and full duplex jamming schemes.

## 1. Introduction

Wireless Sensor Networks (WSN) is a rapidly emerging field and is driven by a wealth of research. In the WSN, sensor nodes collect and process environmental information. Then, the sensor nodes transmit sensed information to a base station. However, data rate of the transmission is limited at low power sensor nodes for a longer battery lifetime. Relaying is a significant technique to increase the data rate for WSN under the power constraint [1,2,3]. Idle sensor nodes with no information to transmit can assist the network by performing as relay nodes. However, the sensor nodes must able to communicate with others sensor nodes. The fifth generation (5G) wireless network, which supports device-to-device communication, will address the demand of inter-sensor communication [4].

Following the broadcast nature of wireless channels, transmission between sensor nodes and base station can be easily overheard and possibly extracted by an eavesdropper. This makes WSN highly susceptible to eavesdropping. In order to achieve a confidential and secure wireless communication, existing systems rely on cryptographic techniques at upper layers [5,6]. However, the cryptographic techniques such as encryption rely on the assumption that the eavesdropper has limited computational capability and is therefore not likely to decipher the key in finite time. Recently, physical layer security has been identified as a promising strategy to provide additional protection against eavesdropping.

Unlike the cryptographic techniques, physical layer security techniques do not rely on computational complexity and will not be compromised by eavesdropper with powerful computational capability. Physical layer security uses wiretap channel coding to achieve the information-theoretic perfect secrecy, where the eavesdropper gains no information about the legitimate information [7,8,9,10]. Physical-layer security exploits the characteristics of the wireless channel to improve transmission security. The secrecy of wireless transmission can be quantified by the secrecy capacity, which is defined as the maximum secrecy rate which can be conveyed to legitimate receiver while the eavesdropper gaining no information about the secrecy message [11]. On the other hand, an intercept event occurs and transmission becomes insecure when the secrecy rate falls below zero. The transmission with lower probability of occurrence of an intercept event, *i.e.*, intercept probability, is more secure and robust against eavesdropping. However, the achievable secrecy rate and intercept probability are severely degraded due to the fading effect of wireless communication. To overcome this limitation, extra cooperative node can be used to improve the secrecy [12,13].

A cooperative node injecting jamming signal to interfere the eavesdropper can improve the secrecy rate. However, the jamming signal may deteriorate the desired legitimate transmission as well. This can be avoided by performing beamforming to minimize the adverse effect of the jamming signal towards the desired data transmission [14]. As a result, the jamming signal consumes additional power resource and the design of beamformer increases the complexity. On the other hand, the cooperative relaying has been identified as a promising technique which not only improves reliability and data rate but also can be further utilized to ensure the secrecy of wireless transmission [15,16,17,18,19,20].

Conventionally, a half-duplex relay cannot perform simultaneous transmission and reception of signal within the same frequency channel. Therefore, when the half-duplex relay is transmitting a signal, the source has to stop transmission. As a result, the spectral efficiency of conventional half-duplex relay is at most half of the spectral efficiency of direct transmission. In order to improve the spectral efficiency of cooperative relaying transmission, a full-duplex relay which can perform simultaneous transmission and reception in the same frequency channel has been proposed. However, in practice, the transmission of the full-duplex relay is interfering its own reception. This self-interference is the main detrimental factor in full-duplex relaying. Since the transmit and receive antennas of full-duplex relay are co-located, the self-interference is much stronger (*i.e.*, 99 dB as reported in [21]) than the intended received signal. The self-interference saturates the analog-to-digital converter (ADC) at the receiver and making it challenging for the cancellation of known self-interference. The suppression of self-interference requires sophisticated hardware and/or advanced signal processing which significantly increase the cost and complexity of relays [22,23,24]. In fact, for full-duplex relaying, the combination of propagation domain, analog domain and digital domain cancellation techniques are needed to achieve good suppression of self interference [25,26]. In [27], full-duplex and full-duplex jamming secrecy network are proposed. Full-duplex relay improves achievable secrecy rate and secrecy outage probability by providing higher spectral efficiency than conventional half-duplex relay. In full-duplex jamming secrecy network, the full-duplex relay transmits jamming signal towards the eavesdroppers while concurrently receives source message, in order to achieve lower secrecy outage probability [27].

Compared to full-duplex relay, the implementation of a half-duplex relay is much simpler and cheaper. Two-path successive relaying (TPSR) has been proposed as an alternative to achieve the full-duplex spectral efficiency by scheduling a pair of half-duplex relays to assist the source transmission alternately [28]. In TPSR, since the two relays are physically separated, the separation distance between relays is able to attenuate the inter-relay interference due to the distance path loss effect. Since the inter-relay interference is much weaker than the self-interference encountered in full-duplex relay with co-located transmit and receive antennas, simple interference management techniques, such as treating the interference as noise or successive interference cancellation, is effective [29,30]. Existing literature mainly considers the TPSR in conventional scenarios without eavesdroppers [31,32,33,34,35]. The performance of TPSR in secrecy communication remains unexplored.

In this paper, we propose a secure TPSR protocol that can provide full-duplex spectral efficiency. Two half-duplex relays are used to forward messages from source to destination alternately, the source transmits new messages continuously, and full-duplex spectral efficiency can be achieved. We evaluate the performance of the proposed protocol in terms of ergodic achievable secrecy rate, intercept probability and secrecy outage probability. The performance is compared with half-duplex relaying, full-duplex relaying and full-duplex jamming schemes in [27]. We also analyze the intercept probability of the proposed scheme and full-duplex relaying.

The contributions of this paper are listed as follows. Firstly, we propose secrecy TPSR which is still unexplored by any existing literature. We evaluate the achievable performance of the proposed secrecy TPSR in terms of ergodic achievable secrecy rate, intercept probability and secrecy outage probability. Secondly, we compare the achievable performance of proposed schemes with the existing half-duplex relaying, full-duplex relaying and full-duplex jamming schemes. Finally, the lower bound intercept probabilities of proposed scheme and existing full-duplex relaying are derived and verified with simulations.

The remaining of the paper is organized as follows. In Section 2, the system model and transmission protocol of TPSR are explained and the intercept probability of TPSR is analyzed in Section 3. In Section 4, the achievable secrecy rate of comparison schemes are presented. In Section 5, the numerical simulations are presented to verify the analysis. Finally, the conclusions is given in Section 6.

## 2. Secrecy Two-Path Successive Relaying Network

### 2.1. System Model

**Figure 1:**
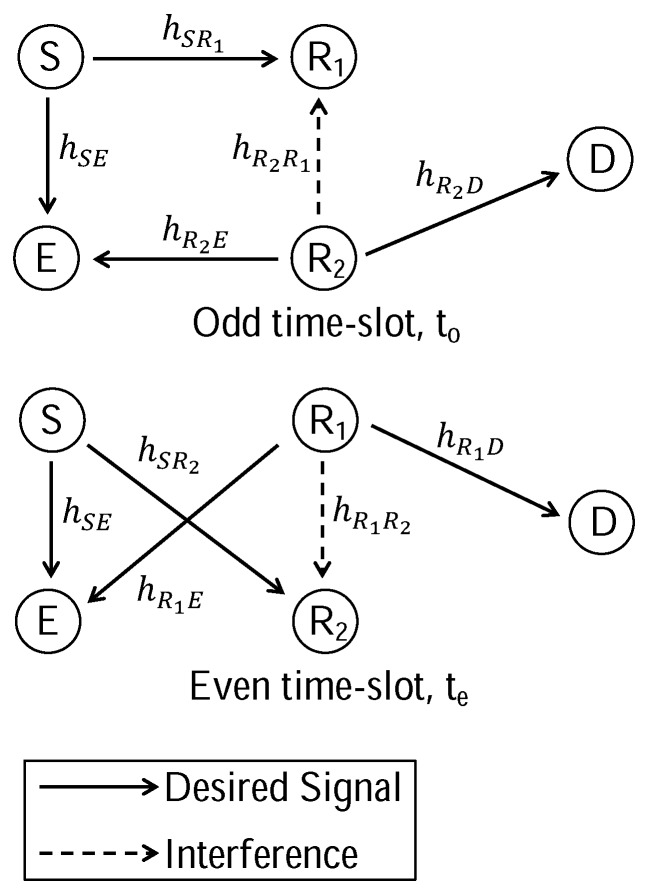
The secrecy two-path successive relaying (TPSR) network with an eavesdropper.

Consider a wireless network consisting of a source (S), a destination (D), two half-duplex relays ($R_{1}$ and $R_{2}$) and an eavesdropper (E) as shown in Figure 1, where all nodes are equipped with a single antenna. The eavesdropper can intercept the transmission from source and relay simultaneously. $R_{1}$ and $R_{2}$ apply the decode-and-forward relaying protocol. It is assumed that the direct S-to-D channel is not available due to severe path loss and/or shadowing. Therefore, the transmission from S to D requires the assistance of $R_{1}$ and $R_{2}$. In additional, the channel-state-information (CSI) for all channels (including the eavesdropping channels) are required for wiretap channel coding.

We assume that all channels experience block Rayleigh fading (Rayleigh fading represents the worst case scenario if compared with the case with strong line of sight and path loss. The results presented in this paper therefore represent the achievable lower bound if compared environment with strong line of sight) and remain constant over one block but vary independently from one block to another. The channel coefficient from node *i* to node *j* is denoted as $h_{ij}$ and channel reciprocity is assumed, *i.e.*, $h_{ij}=h_{ji}$. The corresponding channel gain, ${\left|h_{ij}\right|}^{2}$ are independently exponentially distributed with mean of $\lambda_{ij}$. The noise at relays ($R_{1}$ and $R_{2}$), D and E are denoted as $n_{r_{1}}\left(t\right)$, $n_{r_{2}}\left(t\right)$, $n_{d}\left(t\right)$ and $n_{e}\left(t\right)$ with variances of $\sigma_{r_{1}}^{2}$, $\sigma_{r_{2}}^{2}$, $\sigma_{d}^{2}$ and $\sigma_{e}^{2}$, respectively. The transmit power of source and relays are *P*.

### 2.2. Transmission Protocol

The transmission protocol of two-path successive relaying (TPSR) is divided into T+1 consecutive equal-duration time-slots where S transmits independent codeword $x_{s}\left(t\right),$ t=1,2,…,T continuously. The protocol is alternated by odd time-slot stage ($t_{o}=1,\;3,\;\ldots ,\;T+1$) and even time-slot stage ($t_{e}=2,\;4,\;\ldots ,\;T$) as shown in Figure 1.

- In odd time-slots, S transmits $x_{s}\left(t_{o}\right)$ and $R_{2}$ forwards $x_{s}\left(t_{o}-1\right)$. $R_{1}$ receives $x_{s}\left(t_{o}\right)$ from S while being interfered by $R_{2}$ (inter-relay interference) and D receives $x_{s}\left(t_{o}-1\right)$ from $R_{2}$. E receives both $x_{s}\left(t_{o}\right)$ and $x_{s}\left(t_{o}-1\right)$ simultaneously.
- In even time-slots, S transmits $x_{s}\left(t_{e}\right)$ and $R_{1}$ forwards $x_{s}\left(t_{e}-1\right)$. $R_{2}$ receives $x_{s}\left(t_{e}\right)$ from S while being interfered by $R_{1}$ (inter-relay interference) and D receives $x_{s}\left(t_{e}-1\right)$ from $R_{1}$. E receives both $x_{s}\left(t_{e}\right)$ and $x_{s}\left(t_{e}-1\right)$ simultaneously.

In odd or even time-slot, the eavesdropper jointly decodes messages from the source and relay transmitter. At the same time, the relay receiver is interfered by the inter-relay interference from the transmitting relay. This is similar to the self-interference of full-duplex relay where the transmission from the transmitting antenna interferes the received signal at the receiving antenna. However, the interference mitigation technique of TPSR is simpler than the full-duplex relay since the two relays are physically distributed. The inter-relay interference can be mitigated to the noise floor when the two relays are sufficiently apart [30].

Let $y_{r_{i}}\left(t\right)$, i∈{1,2}, $y_{d}\left(t\right)$ and $y_{e}\left(t\right)$ be the received signals in time slot *t* at $R_{i}$, D and E, respectively. Assume that the relays, destination and eavesdropper can always decode the data. The received signals are

(1) $$y_{r_{i}}\left(t\right)=\sqrt{P}\;h_{SR_{i}}\;x_{s}\left(t\right)+\sqrt{P}\;h_{RR}\;x_{s}\left(t-1\right)+n_{r_{i}}\left(t\right)$$

(2) $$y_{d}\left(t\right)=\sqrt{P}\;h_{R_{i}D\;}x_{s}\left(t-1\right)+n_{d}\left(t\right)$$

(3) $$y_{e}\left(t\right)=\sqrt{P}\;h_{SE}\;x_{s}\left(t\right)+\sqrt{P}\;h_{R_{i}E}\;x_{s}\left(t-1\right)+n_{e}\left(t\right)$$

where $h_{RR}$ is the reciprocal inter-relay channel coefficient.

### 2.3. Achievable Secrecy Rates

From Equations (1) and (2), the channel capacities for S-to-$R_{i}$ and $R_{i}$-to-D are given by

(4) $$\begin{array}{c} C_{sr_{i}}=\frac{1}{2}\;\log_{{}_{2}}\left(1+\frac{P{\left|h_{SR_{i}}\right|}^{2}}{P{\left|h_{RR}\right|}^{2}+\sigma_{r_{i}}^{2}}\right) \end{array}$$

(5) $$\begin{array}{cc} C_{r_{i}d} & =\frac{1}{2}\;\log_{{}_{2}}\left(1+\frac{P{\left|h_{R_{i}D}\right|}^{2}}{\sigma_{d}^{2}}\right) \end{array}$$

respectively. The data transmission rate for $R_{i}$ assisted link is

(6) $$\begin{array}{cc} C_{R_{i}} & =\min\left(C_{sr_{i}},\;C_{r_{i}d}\right) \end{array}$$

On the other hand, the eavesdropper can receive $x_{s}\left(t\right)$ from S and $R_{i}$ at time slot *t* and t+1 respectively as specified in Equation (3). By assuming that the eavesdropper is able to jointly decode the information symbols from S and $R_{i}$, the instantaneous eavesdropping rate (with the use of instantaneous eavesdropping rate, the near-far effect when eavesdropper is located closer to $R_{1}$ than $R_{2}$ can be readily captured by the equation) for $R_{i}$ assisted link can be obtained as

(7) $$\begin{array}{cc} C_{e_{i}} & =\frac{1}{2}\;\log_{{}_{2}}\left(1+\frac{P{\left|h_{SE}\right|}^{2}+P{\left|h_{R_{i}E}\right|}^{2}}{\sigma_{e}^{2}}\right) \end{array}$$

From Equations (6) and (7), the achievable secrecy rate for $R_{i}$ assisted link can be obtained as

(8) $$C_{s_{i}}={\left[C_{R_{i}}-C_{e_{i}}\right]}^{+}$$

where ${\left[x\right]}^{+}=\max\left(x,\;0\right)$.

In TPSR, the $R_{1}$ and $R_{2}$ operate in a time-division mode and forward messages from S to D successively in turn. The achievable secrecy rate for TPSR is the sum of achievable secrecy rate for $R_{1}$ and $R_{2}$ assisted link as follows:

(9) $$C_{{}_{TPSR}}=C_{s_{1}}+C_{s_{2}}$$

## 3. Analysis on Intercept Probability

This section provides the intercept probability analysis of the proposed TPSR. Intercept probability is the probability that the eavesdropper successfully intercepts the transmission signal. The eavesdropper can intercept the transmission signal when the transmission rate of legitimate transmission falls below the eavesdropping rate [36].

**Theorem 1.**  *Assume that the channel gain, ${\left|h_{ij}\right|}^{2}$ of each of the channels is independently exponentially distributed with mean of $\lambda_{ij}$, the intercept probability for $R_{i}$ assisted link can be obtained as follows:*

(10) $$\begin{array}{cc} P_{int_{i}} & =1+\alpha \exp\left(\alpha +\frac{\lambda_{sr_{i}}+\lambda_{r_{i}d}}{\lambda_{{}_{rr}}\lambda_{r_{i}d}}\right)\mathrm{Ei}\left(-\alpha -\frac{\lambda_{sr_{i}}+\lambda_{r_{i}d}}{\lambda_{{}_{rr}}\lambda_{r_{i}d}}\right) \end{array}$$

*where $\alpha =\frac{\lambda_{sr_{i}}}{\lambda_{{}_{rr}}\left(\lambda_{se}+\lambda_{r_{i}e}\right)}$ and $\mathrm{Ei}\left(\cdot \right)$ is the exponential integral function,* i.e.*, $\mathrm{Ei}\left(-x\right)=\int_{x}^{\infty }-\exp\left(-t\right)/t\;dt$.*

**Proof.**  From Equation (8), the intercept probability for $R_{i}$ assisted link can be obtained as:

(11) $$\begin{array}{cc} P_{int_{i}} & =\mathrm{Pr}\left(C_{s_{i}}<0\right)\\ & =\mathrm{Pr}\left(C_{R_{i}}<C_{e_{i}}\right) \end{array}$$

where $C_{R_{i}}$ and $C_{e_{i}}$ are the achievable instantaneous transmission and eavesdropping rates for $R_{i}$ assisted link in Equations (6) and (7), respectively. By using the high signal-to-noise ratio (SNR) approximation, *i.e.*, $\sigma^{2}\to 0$, the $C_{sr_{i}}$ and $C_{r_{i}d}$ in Equation (6) can be approximated as follows:

(12) $$C_{sr_{i}}\approx \frac{1}{2}\;\log_{{}_{2}}\left(\frac{P{\left|h_{SR_{i}}\right|}^{2}}{P{\left|h_{RR}\right|}^{2}+\sigma_{r_{i}}^{2}}\right)$$

(13) $$\begin{array}{cc} C_{r_{i}d} & \approx \frac{1}{2}\;\log_{{}_{2}}\left(\frac{P{\left|h_{R_{i}D}\right|}^{2}}{\sigma_{d}^{2}}\right) \end{array}$$

Then, the $C_{R_{i}}$ can be approximated as follows:

(14) $$\begin{array}{cc} C_{R_{i}} & \approx \frac{1}{2}\;\min\left[\log_{{}_{2}}\left(\frac{P{\left|h_{SR_{i}}\right|}^{2}}{P{\left|h_{RR}\right|}^{2}+\sigma_{r_{i}}^{2}}\right),\;\left(\log_{{}_{2}}\frac{P{\left|h_{R_{i}D}\right|}^{2}}{\sigma_{d}^{2}}\right)\right] \end{array}$$

On the other hand, because the eavesdropper can receive $x_{s}\left(t\right)$ from S and $R_{i}$ at time slot *t* and t+1 respectively, the instantaneous eavesdropping rate with high SNR approximation can be approximated as follows:

(15) $$\begin{array}{cc} C_{e_{i}} & =\frac{1}{2}\;\log_{{}_{2}}\left(1+\frac{P{\left|h_{SE}\right|}^{2}+P{\left|h_{R_{i}E}\right|}^{2}}{\sigma_{e}^{2}}\right)\\ & \approx \frac{1}{2}\;\log_{{}_{2}}\left(\frac{P{\left|h_{SE}\right|}^{2}+P{\left|h_{R_{i}E}\right|}^{2}}{\sigma_{e}^{2}}\right) \end{array}$$

by ignoring the interference of simultaneous transmission from the S and $R_{i}$. Substituting Equations (14) and (15) into Equation (11) produces the intercept probability for $R_{i}$ assisted link as follows:

(16) $$\begin{array}{cc} P_{int_{i}} & =\mathrm{Pr}\left\{\min\left[\log_{{}_{2}}\left(\frac{P{\left|h_{SR_{i}}\right|}^{2}}{P{\left|h_{RR}\right|}^{2}+\sigma_{r_{i}}^{2}}\right),\;\log_{{}_{2}}\left(\frac{P{\left|h_{R_{i}D}\right|}^{2}}{\sigma_{d}^{2}}\right)\right]<\log_{{}_{2}}\left(\frac{P{\left|h_{SE}\right|}^{2}+P{\left|h_{R_{i}E}\right|}^{2}}{\sigma_{e}^{2}}\right)\right\} \end{array}$$

From Equation (16), the intercept probability for $R_{i}$ assisted link can be obtained as follows:

(17) $$\begin{array}{cc} P_{int_{i}} & =\int_{0}^{\infty }\int_{0}^{y}\;f_{{}_{X}}\left(x\right)\;f_{{}_{Y}}\left(y\right)\;dx\;dy\\ & =\int_{0}^{\infty }f_{Y}\left(y\right)\;F_{X}\left(y\right)\;dy \end{array}$$

where $f_{{}_{X}}\left(x\right)$ and $f_{{}_{Y}}\left(y\right)$ are the probability density function (PDF) of $X=\min\left(\frac{P{\left|h_{SR_{i}}\right|}^{2}}{P{\left|h_{RR}\right|}^{2}+\sigma_{r_{i}}^{2}},\;\frac{P{\left|h_{R_{i}D}\right|}^{2}}{\sigma_{d}^{2}}\right)$ and $Y=\frac{P{\left|h_{SE}\right|}^{2}+P{\left|h_{R_{i}E}\right|}^{2}}{\sigma_{e}^{2}}$, respectively, and $F_{{}_{X}}\left(y\right)$ is the cumulative distribution function (CDF) of *X*.

The $f_{{}_{Y}}\left(y\right)$ and $F_{{}_{X}}\left(y\right)$ (see [27]) can be obtained as,

(18) $$f_{{}_{Y}}\left(y\right)=\frac{1}{\lambda_{se}+\lambda_{r_{i}e}}\exp\left(-\frac{y}{\lambda_{se}+\lambda_{r_{i}e}}\right)$$

and

(19) $$\begin{array}{cc} F_{{}_{X}}\left(y\right) & =1-\frac{\lambda_{sr_{i}}}{\lambda_{sr_{i}}+\lambda_{rr}y}\exp\left(-\frac{y\left(\lambda_{sr_{i}}+\lambda_{r_{i}d}\right)}{\lambda_{sr_{i}}\lambda_{r_{i}d}}\right) \end{array}$$

respectively. By using (3.352.4) in [37], the intercept probability for $R_{i}$ assisted link in Equation (17) is solved and shown in Equation (10). ☐

**Remark 1**  (Remark of Theorem 1). *Theorem 1 shows the closed form intercept probability for $R_{i}$ assisted link in TPSR. From Equation (10), the interception probability for $R_{i}$ assisted link varies with the mean of corresponding channel gain,* i.e.*, $\lambda_{ij}$. By having higher $\lambda_{sr_{i}}$ and $\lambda_{r_{i}d}$ for $h_{SR_{i}}$ and $h_{R_{i}D}$, respectively, the interception probability for $R_{i}$ assisted link is decreased. On the other hand, the $\lambda_{{}_{rr}}$, $\lambda_{se}$ and $\lambda_{r_{i}e}$ for $h_{RR}$, $h_{SE}$ and $h_{R_{i}E}$, respectively, are the degrading factors, which increases the probability of eavesdropper to intercept the transmission signal.*

**Corollary 2.**  *Intercept probability of TPSR is lower bounded by*

(20) $$\begin{array}{cc} P_{{}_{TPSR-L}} & =P_{int_{1}}P_{int_{2}}\\ & =\mathrm{Pr}\left(C_{s_{1}}<0\right)\mathrm{Pr}\left(C_{s_{2}}<0\right) \end{array}$$

**Proof.**  The intercept probability of TPSR is

(21) $$P_{{}_{TPSR}}=\mathrm{Pr}\left(C_{{}_{TPSR}}<0\right)$$

where $C_{{}_{TPSR}}=C_{s_{1}}+C_{s_{2}}$. The closed form of $P_{{}_{TPSR}}$ is not achievable due to complexity of derivation. Alternately, lower bound of $P_{{}_{TPSR}}$ can be obtained based on Theorem 1. Theorem 1 shows the closed form intercept probability for $R_{1}$ and $R_{2}$ assisted links in TPSR. The transmission and reception of $R_{1}$ and $R_{2}$ are mutually independent. Thus, the lower bound intercept probability of TPSR, $P_{{}_{TPSR-L}}$ is the product of intercept probability for $R_{1}$ and $R_{2}$ assisted link as shown in Equation (20). ☐

**Remark 2**  (Remark of Corollary 1). *Corollary 1 in Equation (20) shows that intercept probability of TPSR is lower bounded by the product of intercept probability for $R_{1}$ and $R_{2}$ assisted link. Meanwhile, the intercept probability of full-duplex relaying (FDR) is the intercept probability for a full-duplex relay (FR) assisted link as shown in Equation (26). Equations (20) and (26) reveal that the lower bound intercept probability of TPSR is quadratically lower than the FDR. This is because of the two mutually independent assisted link of $R_{1}$ and $R_{2}$ in TPSR.*

## 4. Comparable Schemes

In this section, we review the achievable secrecy rate of half-duplex, full-duplex and full-duplex jamming secrecy network in [27].

### 4.1. Secrecy Half-Duplex Relaying Network

Half-duplex relaying (HDR) is a conventional relay scheme with a half-duplex relay, R. In the HDR scheme, S transmits $x_{s}\left(t\right)$ to R in time-slot *t* and R forwards $x_{s}\left(t\right)$ to D in subsequent time-slot. During the transmission, E receives $x_{s}\left(t\right)$ twice, from S and R at time slot *t* and t−1, respectively.

The data transmission rate for HDR is

(22) $$\begin{array}{cc} C_{R} & =\frac{1}{2}\;\min\left[\log_{{}_{2}}\left(1+\frac{P{\left|h_{SR}\right|}^{2}}{\sigma_{r}^{2}}\right),\log_{{}_{2}}\left(1+\frac{P{\left|h_{RD}\right|}^{2}}{\sigma_{d}^{2}}\right)\right] \end{array}$$

On the other hand, the eavesdropping rate can be obtained as

(23) $$C_{e}=\frac{1}{2}\;\log_{{}_{2}}\left(1+\frac{P{\left|h_{SE}\right|}^{2}+P{\left|h_{RE}\right|}^{2}}{\sigma_{e}^{2}}\right)$$

Then, the achievable secrecy rate of HDR is given by

(24) $$C_{{}_{HDR}}={\left[C_{R}-C_{e}\right]}^{+}$$

### 4.2. Secrecy Full-Duplex Relaying Network

The system model of full-duplex secrecy relay network in [27] is similar to the half-duplex secrecy relay network, except now the relay has two antennas for simultaneous receiving and transmission respectively, *i.e.*, full-duplex relay, FR. With the two antennas, FR can receive $x_{s}\left(t\right)$ from S and forward the previously decoded $x_{s}\left(t-1\right)$ to D simultaneously at time slot *t*. Therefore, S and D can transmit and receive continuously. However, when FR is receiving $x_{s}\left(t\right)$ from S, it is interfered by its own transmission known as self-interference.

The data transmission rate and eavesdropping rate for full-duplex relaying (FDR) are two times of Equations (6) and (7) respectively and $h_{RR}$ is the self-interference channel coefficient of FR. Then, the achievable secrecy rate of FDR is given by

(25) $$\begin{array}{cc} C_{{}_{FDR}} & ={\left[\min\left(2\;C_{sr_{i}},\;2\;C_{r_{i}d}\right)-\frac{1}{T}\;\log_{{}_{2}}\left(\det\left\{I+H_{e}^{H}H_{e}\right\}\right)\right]}^{+} \end{array}$$

Based on the intercept probability analysis in Section 3, the interception probability of FDR is the same as the intercept probability of $R_{1}$ or $R_{2}$ assisted link in TPSR as follows (see Equation (10))

(26) $$\begin{array}{cc} P_{{}_{FDR}} & =P_{int_{i}}\\ & =\mathrm{Pr}\left(C_{s_{i}}<0\right) \end{array}$$

### 4.3. Secrecy Full-Duplex Jamming Network

The full-duplex jamming (FDJ) network is proposed in [27]. During time slot *t*, S transmits $x_{s}\left(t\right)$ to FR and FR receives and decodes $x_{s}\left(t\right)$ from S while transmitting a jamming signal to E. During time slot t+1, FR forwards previously decoded $x_{s}\left(t\right)$ to D and switches off its receiving antenna. At the same time, S transmits jamming signal to E.

The data transmission rate for FDJ is given in Equation (6) and $h_{RR}$ is the self-interference channel coefficient of FR. On the other hand, the eavesdropping rate for FDJ is given by

(27) $$C_{e}=\frac{1}{2}\log_{{}_{2}}\left(1+\frac{P{\left|h_{SE}\right|}^{2}}{P{\left|h_{RE}\right|}^{2}+\sigma_{e}^{2}}+\frac{P{\left|h_{RE}\right|}^{2}}{P{\left|h_{SE}\right|}^{2}+\sigma_{e}^{2}}\right)$$

Then, the achievable secrecy rate of FDJ can be obtained as

(28) $$\begin{array}{cc} C_{{}_{FDJ}} & ={\left[\min\left(C_{sr_{i}},\;C_{r_{i}d}\right)-C_{e}\right]}^{+} \end{array}$$

## 5. Numerical Results

In this section, several Monte Carlo simulation results of the proposed two-path successive relaying (TPSR) and existing half-duplex relaying (HDR), full-duplex relaying (FDR) and full-duplex jamming (FDJ) schemes are presented. In the simulations, the transmit power of source and relay, *P* is fixed to unity and the SNR for channel from node *i* to node *j* is defined as $\gamma_{{}_{ij}}=1/\sigma_{j}^{2}$. There are T=1000 independent codewords transmitted from the source in all schemes. In this paper, we assume that the self-interference is the residual self-interference [27] after the self-interference suppression, which has the same level as the receiver noise. For fair comparison, we assume that the inter-relay interference is at noise level, which can be achieved through physical separation between the relays, relay selection, other techniques, *etc*.

**Figure 2:**
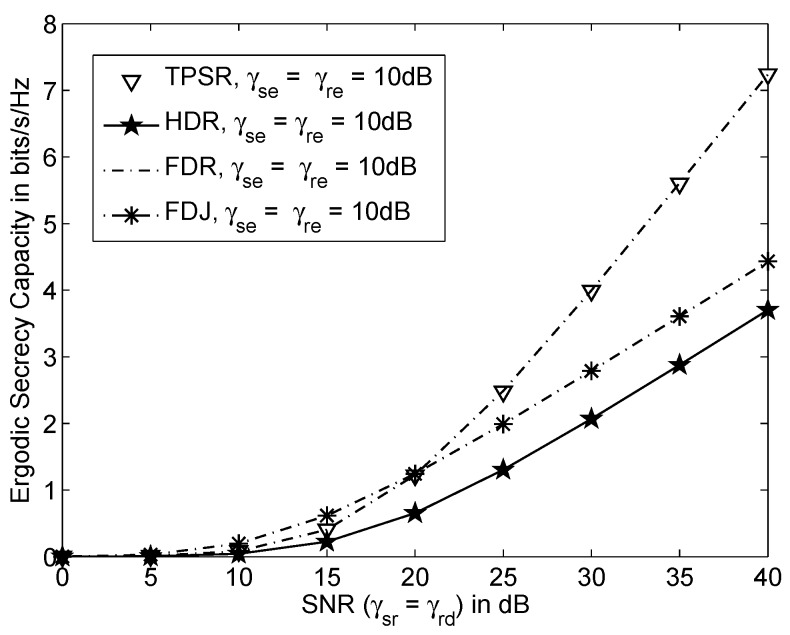
Ergodic achievable secrecy rate *versus* SNR where $\gamma_{sr}=\gamma_{rd}$, $\gamma_{se}=\gamma_{re}=10\;\mathrm{dB}$ and the inter-relay interference or self-interference, $\gamma_{rr}=0\;\mathrm{dB}$.

Figure 2 shows the ergodic achievable secrecy rate *versus* SNR of various schemes when $\gamma_{sr}=\gamma_{rd}$, $\gamma_{se}=\gamma_{re}=10\;\mathrm{dB}$ and the inter-relay interference or residual self-interference, $\gamma_{rr}=0\;\mathrm{dB}$. It is obvious that TPSR and FDR achieved the same ergodic achievable secrecy rate. This means that the TPSR has the same bandwidth efficiency as the FDR. The TPSR and FDR also achieved 95.4% and 63.3% ergodic secrecy rate gain compared to HDR and FDJ, respectively, when SNR=40dB. This is because the higher bandwidth efficiency of TPSR and FDR compared to the HDR and FDJ. The FDJ employs jamming technique to interfere the eavesdropper. As a result, the FDJ achieves higher ergodic achievable secrecy rate than the HDR. However, FDJ achieves a lower ergodic achievable secrecy rate than TPSR and FDR because half of the bandwidth is used to transmit jamming signals.

**Figure 3:**
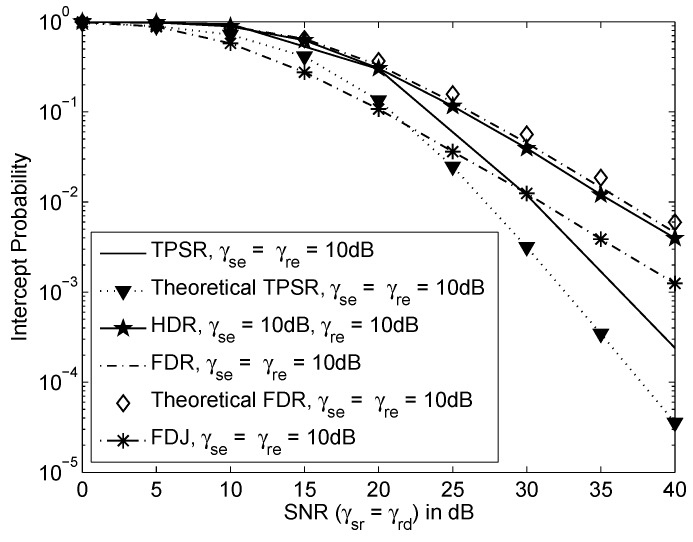
Intercept probability *versus* SNR where $\gamma_{sr}=\gamma_{rd}$, $\gamma_{se}=\gamma_{re}=10\;\mathrm{dB}$ and the inter-relay interference or self-interference, $\gamma_{rr}=0\;\mathrm{dB}$.

Figure 3 shows the intercept probability of various schemes *versus* SNR when $\gamma_{sr}=\gamma_{rd}$, $\gamma_{se}=\gamma_{re}=10\;\mathrm{dB}$ and the inter-relay interference or residual self-interference, $\gamma_{rr}=0\;\mathrm{dB}$. We observe that the FDR has higher probability of interception compared to the HDR. This is because of the residual self-interference of full-duplex relay in FDR. By transmitting jamming signal to the eavesdropper, the FDJ achieves lower probability of interception compared to the FDR and HDR. The intercept probability of TPSR is lower bounded by theoretical result. Meanwhile, the theoretical result of FDR are well matched to the simulation result. This verifies that the lower bound intercept probability of TPSR and FDR in Equations (20) and (26), respectively. TPSR also achieves the lowest probability of interception compared to all the other schemes at high SNR, *i.e.*, SNR≥30dB. This is because the two mutually independent assisted link of $R_{1}$ and $R_{2}$ contribute lower intercept probability to the TPSR compared to the other schemes equipped with only one relay.

**Figure 4:**
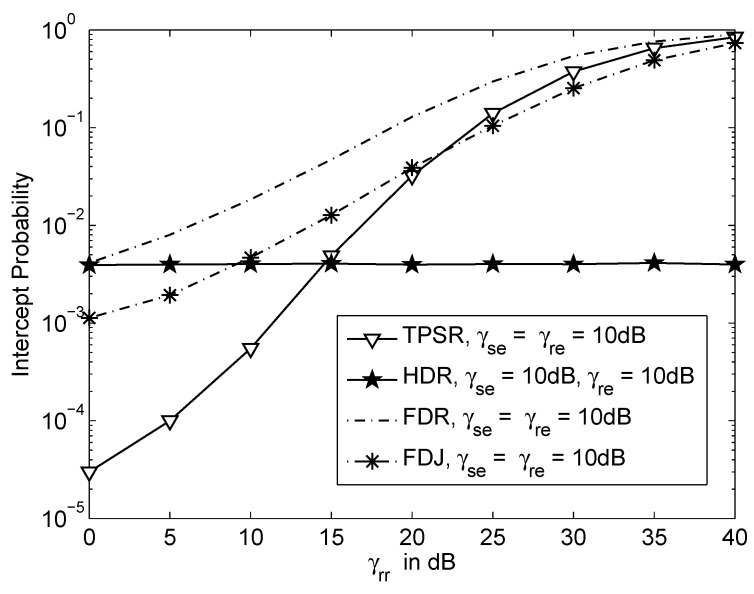
Intercept probability *versus* inter-relay interference or residual self-interference, $\gamma_{rr}$ where $\gamma_{sr}=\gamma_{rd}=40\;\mathrm{dB}$ and $\gamma_{se}=\gamma_{re}=10\;\mathrm{dB}$.

Figure 4 shows the intercept probability *versus* inter-relay interference or residual self-interference, $\gamma_{rr}$ for various schemes when $\gamma_{sr}=\gamma_{rd}=40\;\mathrm{dB}$ and $\gamma_{se}=\gamma_{re}=10\;\mathrm{dB}$. The FDR has the highest probability of interception compared to the other schemes even when $\gamma_{rr}=0\;\mathrm{dB}$. The residual self-interference decreases the data transmission rate of the FDR. Therefore, the FDR has higher probability of interception compared to the HDR. However, by employing jamming technique, the FDJ with low residual self-interference can achieve lower probability of interception compared to the HDR. On the other hand, when the inter-relay interference $\gamma_{rr}<15\;\mathrm{dB}$, the TPSR has the lowest probability of interception compared to the other schemes. This shows that the operating requirement for inter-relay interference level in TPSR is much lower and practical if compared to the self interference level in FDR.

**Figure 5:**
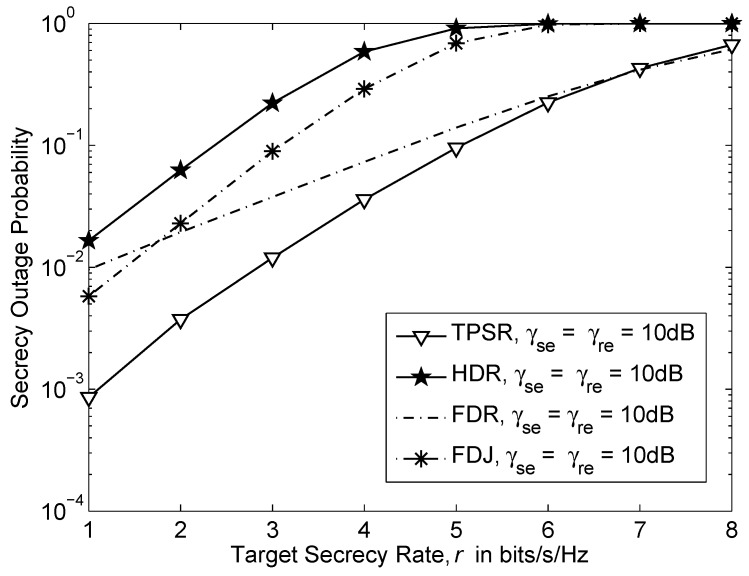
Secrecy outage probability *versus* target secrecy rate, *r* where $\gamma_{sr}=\gamma_{rd}=40\;\mathrm{dB}$, $\gamma_{se}=\gamma_{re}=10\;\mathrm{dB}$ and the inter-relay interference or self-interference, $\gamma_{rr}=0\;\mathrm{dB}$.

Figure 5 shows the secrecy outage probability *versus* target secrecy rate, *r* of various schemes when $\gamma_{sr}=\gamma_{rd}=40\;\mathrm{dB}$, $\gamma_{se}=\gamma_{re}=10\;\mathrm{dB}$ and the inter-relay interference or residual self-interference, $\gamma_{rr}=0\;\mathrm{dB}$. The probability of secrecy outage of all the schemes is increasing when the target secrecy rate, *r* is increased. The TPSR has the lowest probability of secrecy outage compared to the other schemes and it is lower bounded by the FDR. This is due to the use of two relays in TPSR which provide additional diversity and full-duplex bandwidth efficiency. In contrast to previous results in Figure 3, where the FDR has lower probability of secrecy outage than the HDR and FDJ. This is because the “1/2” pre-log factor in achievable secrecy rate of HDR and FDJ in Equations (24) and (28). The jamming technique benefits the FDJ by delivering lower probability of secrecy outage than HDR.

**Figure 6:**
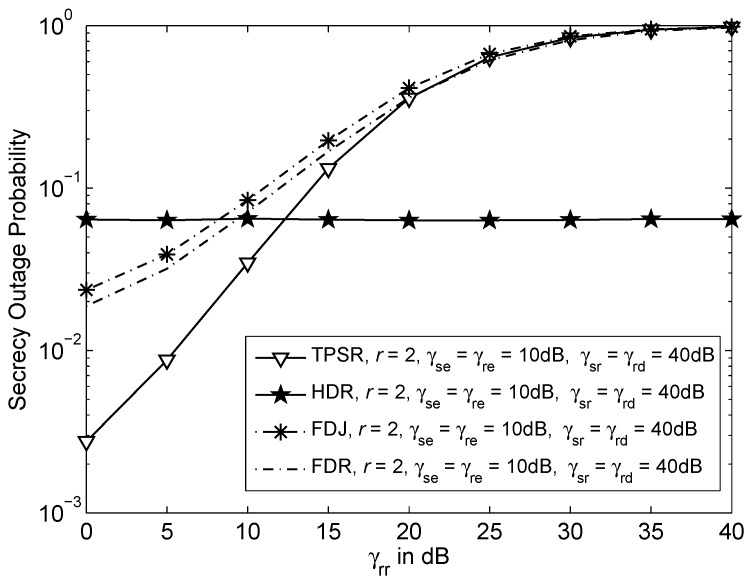
Outage probability *versus* inter-relay interference or self-interference, $\gamma_{rr}$ where target secrecy rate, r=2bits/s/Hz, $\gamma_{se}=\gamma_{re}=10\;\mathrm{dB}$ and $\gamma_{sr}=\gamma_{rd}=40\;\mathrm{dB}$.

Figure 6 shows the secrecy outage probability *versus* inter-relay interference or self-interference, $\gamma_{rr}$ for various schemes when target secrecy rate, r=2bits/s/Hz, $\gamma_{se}=\gamma_{re}=10\;\mathrm{dB}$ and $\gamma_{sr}=\gamma_{rd}=40\;\mathrm{dB}$. The TPSR has the lowest probability of secrecy outage compared to the FDR and FDJ. In other words, with the same $\gamma_{rr}$, TPSR is more secure than the FDR and FDJ. By considering the HDR as baseline scheme, when $\gamma_{rr}=10\;\mathrm{dB}$, the TPSR has lower probability of secrecy outage, whereas the FDR and FDJ have higher probability of secrecy outage. This shows that the FDR and FDJ require much lower $\gamma_{rr}$ compared to the TPSR to achieve lower probability of secrecy outage than the HDR. As a result, the FDR and FDJ have a stricter requirement on residual interference compared to the TPSR.

## 6. Conclusions

In this paper, two-path successive relaying (TPSR) is proposed to improve the security of wireless transmission. We compare the ergodic achievable secrecy rate, interception probability and secrecy outage probability of the proposed TPSR against the existing half-duplex relaying (HDR), full-duplex relaying (FDR) and full-duplex jamming (FDJ). The numerical results reveal that the proposed TPSR achieves the same ergodic achievable secrecy rate as the FDR. The TPSR also delivers the lowest probability of interception and secrecy outage compared to the other schemes due to the full-duplex bandwidth efficiency and the two mutually independent links assisted by relay pair $R_{1}$ and $R_{2}$. The intercept probabilities of TPSR and FDR are analyzed and verified with simulation. The analysis shows that the intercept probability of TPSR is quadratically lower than the intercept probability of FDR. The numerical results show the TPSR has the lowest probability of interception compared to all other schemes when the inter-relay interference, $\gamma_{rr}<15\;\mathrm{dB}$. In terms of secrecy outage probability, the FDR and FDJ demand lower interference level compared to the TPSR in order to outperform the HDR. In short, with the proposed TPSR protocol, a secured wireless transmission can be achieved by using conventional half-duplex relays without employing sophisticated jamming and/or interference cancellation techniques.
